# Does the Presence of Transposable Elements Impact the Epigenetic Environment of Human Duplicated Genes?

**DOI:** 10.3390/genes10030249

**Published:** 2019-03-26

**Authors:** Romain Lannes, Carène Rizzon, Emmanuelle Lerat

**Affiliations:** 1 Laboratoire de Biométrie et Biologie Evolutive UMR 5558, Université de Lyon, Université Lyon 1, CNRS, F-69622 Villeurbanne, France; romain.lannes@gmail.com; 2 Laboratoire de Mathématiques et Modélisation d’Evry (LaMME), Université d’Evry Val d’Essonne, UMR CNRS 8071, ENSIIE, USC INRA, 23 bvd de France, 91037 Evry CEDEX Paris, France; carene.rizzon@univ-evry.fr

**Keywords:** transposable elements, gene duplication, gene evolution, epigenetics

## Abstract

Epigenetic modifications have an important role to explain part of the intra- and inter-species variation in gene expression. They also have a role in the control of transposable elements (TEs) whose activity may have a significant impact on genome evolution by promoting various mutations, which are expected to be mostly deleterious. A change in the local epigenetic landscape associated with the presence of TEs is expected to affect the expression of neighboring genes since these modifications occurring at TE sequences can spread to neighboring sequences. In this work, we have studied how the epigenetic modifications of genes are conserved and what the role of TEs is in this conservation. For that, we have compared the conservation of the epigenome associated with human duplicated genes and the differential presence of TEs near these genes. Our results show higher epigenome conservation of duplicated genes from the same family when they share similar TE environment, suggesting a role for the differential presence of TEs in the evolutionary divergence of duplicates through variation in the epigenetic landscape.

## 1. Introduction

Epigenetic changes can explain part of the variation in gene expression observed between tissues from the same organism [1,2,3,4], or the fate of individuals like in honeybees by affecting the differentiation between the queen and the workers [5] or in the determination of the different casts in ants [6]. These examples are likely to represent only a tiny fraction of all the possible effects of epigenetic processes. In sum, epigenetic modifications are important actors of the gene expression modulation such as variation in expression among tissues, developmental stages or in response to environmental changes [7]. Three epigenetic mechanisms have been identified that can work jointly to regulate gene expression. DNA methylation usually occurs in the context of CpG dinucleotides and is associated with transcription silencing [8,9,10,11]. RNA interference mechanism is characterized by the synthesis of small noncoding RNAs, which, when associated with a protein complex, can target messenger RNAs and trigger their degradation [12,13]. Histone modifications correspond to post-translational biochemical changes occurring at particular amino acid residues of these proteins that are at the basis of nucleosomes [11,14,15]. According to the type of histone modifications, the effect can either compact or relax the chromatin structure; both have a direct impact on the gene expression [3,16]. Due to their important role in gene regulation, epigenetic modifications can potentially cause diseases under certain circumstances when a global modification of the epigenetic landscape happens [17].

It has long been suspected that changes in gene regulation may play a role in the adaptation and evolution of organisms [18]. In particular, epigenetic divergence has been proposed to affect species divergence by conferring hybrid incompatibility like in the example of the formation of mouse subspecies, which is linked to methylation of lysine 4 from histone 3 (H3K4me) [19]. In three cell lines, variation of gene expression in primates could be associated with changes in H3K4me3 localization [20]. Similarly, changes in DNA methylation have been shown to partly explain the divergence of gene expression in the brains of humans and chimps [21]. This variation in DNA methylation could also explain the evolution of vulnerability to some diseases in humans since among the list of impacted genes, several of them have been associated with human diseases like neurodevelopmental and psychological disorders. Epigenetic conservation or divergence is also linked to the DNA sequence conservation. For example, in humans, hypomethylated CpG islands have been shown to be under selective constraints [22]. These CpG islands were also shown to be more enriched in trimethylated H3K4 and H3K36, and in acetylated H3K27 [23]. The acquisition of hypermethylated DNA in humans is coupled to a very rapid nucleotidic evolution near CpG sites [24]. In this last work, the authors showed a genome-wide conservation of DNA methylation profiles when comparing humans and various primates, with the presence of regions with human specific patterns not localized near transcription start sites. Some epigenetic modifications can be conserved between species. For example, the trimethylated H3K36 modification is conserved in exons and introns between humans and mice [25]. A wide comparison of three histone modifications among several cell types from humans and mice showed a strong association between the stability among the cell types (intraspecies) and the conservation between species of these modifications against both genetic and environmental changes [26]. Among invertebrates, gene body DNA methylation has been shown to be conserved on very long evolutionary time scales, suggesting a function of DNA methylation in the different genomes [27]. The same kind of results have been observed in plants in which a strong conservation of gene body methylation was observed that targeted slowly evolving genes, indicating that the methylation level can have evolutionary consequences [28]. At an intraspecific level, epigenetic modifications may be implicated in functional divergence by facilitating tissue-specific regulation. For example, human duplicated genes are initially highly methylated, then gradually lose DNA methylation as they age [29]. Within each pair of genes, DNA methylation divergence increases with time. Moreover, tissue-specific DNA methylation of duplicates correlates with tissue-specific expression, implying that DNA methylation could be a causative factor for functional divergence of duplicated genes [29]. However, epigenetic modifications may also play a role in the functional conservation. For example, in some plants, paralogous genes associated with trimethylated H3K27 showed the highest coding sequence divergence but the highest similarity in expression patterns and in regulatory regions when compared to paralogous genes in which only one gene was the target of this histone modification [30]. In this case, the histone modification could be responsible for the conservation of gene expression. By comparing segmental duplications regions in humans, a widespread conservation of DNA methylation and some histone modifications was observed when considering recently duplicated regions [31]. For the regions displaying divergence in DNA methylation and chromatin states, particular DNA motifs were detected.

Eukaryotic genomes are formed from a variety of elements among which protein-coding genes are a minority. In the human genome, for example, the protein-coding genes represent only a very small fraction (<2% of the genome), whereas repetitive sequences represent more than half [32]. While the non-coding part was first thought to have no function [33], it is now known to be composed of a mixture of repetitive DNA and non-functional sequences interspersed with non-coding RNA genes and regions that are crucial for transcriptional and post-transcriptional regulation [34,35]. The greater part of repeated DNA is classified as transposable elements (TEs), with several millions of them inserted throughout the human genome. Because of their presence in genomes, TEs have a significant impact on genome evolution and on gene functioning [36,37]. For example, a bias in the distribution of TEs in and near genes has been observed, showing that TEs are found to be under represented inside genes, which indicates that they are counter selected in these regions [34,38]. Moreover, TEs have been shown to be associated with the evolution of duplicated genes [39,40]. To counteract their deleterious effects, TEs are regulated by the host genome via epigenetic mechanisms to suppress or silence their activity [41,42]. In normal mammalian cells, TEs are usually methylated, therefore transcriptionally silenced [41]. In some abnormal cells where DNA methylation is abolished, TEs can be mobilized, resulting in a potential impact on the integrity of the cell [43,44]. A change in the local epigenetic landscape associated with the presence of TE sequences is expected to affect the expression of the neighboring genes since these modifications occurring at TE sequences can spread to neighboring sequences, as has been observed in mice, in plants or in fungi [45,46,47,48,49,50,51]. In humans, the recent insertion of an Alu element was identified as the cause of increasing levels of DNA methylation in its surrounding genomic area, which inactivated the neighboring gene expression [52]. When comparing histone modification of genes between normal and cancer conditions in humans, we found that the presence of TEs near genes was associated with more changes in histone enrichment [53]. In primates, some TEs have been identified as a source of novelty in gene regulation, in association with changes in histone modifications [54]. Alu elements were observed to be enriched around methylated sites of discordant paralogous regions corresponding to segmental duplications in human [31]. Differentially methylated regions between humans and primates were shown to be enriched in endogenous retroviruses in hypomethylated human specific regions [24]. Thus, the presence of TEs in a genome may have a direct influence on the epigenetic variations directed on the host genes, potentially influencing their fate and functioning.

In this work, we have explored how the epigenetic modifications of genes are conserved and what the role of TEs is in this conservation. For that, we have studied the conservation of the epigenome at an intraspecific level in humans. By measuring, in different cell types, the divergence of epigenetic modifications associated with duplicated genes and linked to the presence of TEs near the genes, we have determined the impact of TEs on epigenetic changes and expression divergence associated with the time since duplication. Our results show that the presence of TEs is associated with variation in histone modification enrichment and methylation level of neighboring genes but also that a similar TE environment near duplicated genes is related to higher conservation of epigenetic modification and expression.

## 2. Material and Methods

### 2.1. Duplicated Genes

Gene families were retrieved from the HOGENOM database [55], which contains functional proteins from 1400 organisms grouped by sequence homology coming from various nucleotide sequence collections. Among the 10,064 gene families for which we were able to identify Ensembl gene access numbers in the human genome version GRCh38, 1420 families contain two functional human genes (list provided as Supplementary data S1). We determined for each of these pairs the divergence between the two genes by aligning the protein sequences and subsequently the nucleotidic sequences to keep the codon alignments. The sequence divergence estimates between duplicated genes of a given family were computed using the YN00 module of paml [56] to obtain the synonymous substitution rate (dS), the non synonymous substitution rate (dN) and the omega ratio (dN/dS).

### 2.2. Epigenetic Modification and Expression Data

This study makes use of data generated by the BLUEPRINT Consortium (www.blueprint-epigenome.eu). We have retrieved epigenetics and expression data from four normal cell types extracted from cord blood of female individuals and corresponding to two precursor cell types (cd14+cd16− (access number C005PS) and erythroblast (access number S002S3)) and to two differentiated cell types (macrophage (access number S00BHQ) and cd8T (access number C0066P)). Methylation, histone modification and expression data have been generated by the alignment of BS-seq, ChIP-seq and RNA-seq reads on the human genome (version GRCh38) using the mapper bwa [57] with a random location assignment for multiple hits [58]. We thus used the methylation status (hypomethylated, hypermethylated, and standard), and the histone enrichment for six histone modifications (H3K27me3, H3K9me3, H3K27ac, H3K4me1, H3K4me3 and H3K36me3) of genomic regions, and the expression level of annotated genes (FPKM) as provided by the BLUEPRINT Consortium. The mean histone enrichment was computed for each gene and corresponds to the average fold enrichment of the given histone modification for the positions covered by the gene, normalized by the gene size [53]. We have determined the mean level of methylation of each gene from the identified hyper- and hypo-methylated regions covering the gene. Hyper-methylated regions correspond to regions with an average methylation level of >0.75 and hypo-methylated regions have an average methylation level of <0.25. These values correspond to the ratio of reads with an unconverted cytosine (i.e., C) over the sum of all reads containing either an unconverted cytosine or a converted cytosine (i.e., T). We thus have considered a gene globally hypo- or hyper-methylated when the average methylation ratio covering its position was <0.25 or >0.75 respectively. Its level of methylation was considered as standard otherwise. For the expression analysis, a gene was considered as expressed if it has an FPKM value of at least 0.5 [59]. As recommended [60], the expression data of a given gene *i* in each cell type were converted from FPKM to TPM using the formula $TPM_{i}=\frac{FPKM_{i}}{\sum^{\text{​}}FPKM}\cdot 10^{6}$ to normalize the values in each cell type allowing direct comparisons. The divergence of expression between the two genes g1 and g2 from a given family was estimated by the Manhattan distance d_m_ across the four samples according to the formula:

$$d_{m}=\frac{1}{2}\overset{4}{\underset{k=1}{\sum }}\left|\frac{g_{1,k}}{\sum_{k=1}^{4}g_{1,k}}-\frac{g_{2,k}}{\sum_{k=1}^{4}g_{2,k}}\right|$$

### 2.3. Transposable Elements Neighborhood

The TE annotation from the latest version of the human genome assembly was obtained by parsing the repeat-masker output file available on the website of the University of California, Santa Cruz (http://hgdownload.cse.ucsc.edu/goldenPath/hg38/bigZips/) using the program one-code-to-find-them-all [61] with the *--strict* option to avoid false positive identification. This program assembles each TE copy and determine their positions in the genome. Although polymorphic TE insertions are present when comparing different individuals and may locally have an important impact on health, they represent only thousand of insertions, which is fare less than the millions of fixed ones [62]. In this work, we are investigating the influence of fixed TE insertions for normal conditions. For each human coding gene, we computed the TE density and the TE coverage using a 2kb-flanking region upstream and downstream the gene as proposed by Grégoire et al. [53] to cover the promoter region of the genes in addition to the entire gene. The density estimates the number of TEs in a given region normalized by the size of the region and the coverage measures the proportion of nucleotides belonging to an TE in the considered region. We have considered in our approach all types of TEs globally, without differentiating the classes. It is known that epigenetic modifications may differ according to the type of TEs [63]; however, it would be impossible to have a large enough sample size of duplicated genes if considering only those with just one type of TE in their vicinity, the unique condition to really analyze the TE type contribution without any confounding factors due to the presence of other TEs.

### 2.4. Gene Classification

All human coding genes (18,938 genes) were clustered according to their level of density and coverage of TEs using the K-medoids algorithm as implemented in the pam() function of the R package [64], which allows an unsupervised classification in a defined number of classes. We thus defined five gene categories from TE-free genes (genes with no TE in their neighborhood) to TE-very-rich genes (genes with numerous TE in their neighborhood). The genes with density and coverage of 0 were defined as TE-free genes. The remaining genes were clustered using both density and coverage values to discriminate between the TE-very-poor (mean density of 0.0003 insertions/pb and mean coverage of 0.086), TE-poor (mean density of 0.0007 insertions/pb and mean coverage of 0.196), TE-rich (mean density of 0.0012 insertions/pb and mean coverage of 0.304), and TE-very-rich genes (mean density of 0.0025 insertions/pb and mean coverage of 0.419).

We determined three age classes (young, middle-age and old) of gene families based on the intra family synonymous substitution rate (dS) values with young families corresponding to gene pairs with dS < 1, middle-age families corresponding to gene pairs with 1 ≤ dS < 2, and old families corresponding to gene pairs with dS > 2 [29].

### 2.5. Statistical Tests

All statistical analyses were performed using R version 3.2.3 [64]. The Kolmogorov-Smirnov test was used to compare the distribution of two samples, the Kruskall-Wallis test was used to determine whether samples originated from the same distribution, and the Spearman test was used to determine if the correlations between the compared data were significantly not null. The Pearson’s chi-squared goodness of fit test was used to determine whether there was a significant difference between the expected and the observed frequencies in one or more categories of possible associations of TE context for duplicated gene pairs. It is designed to test the null hypothesis that an observed frequency distribution is consistent with a hypothesized theoretical distribution. *P*-values were computed by Monte Carlo simulations with 2000 replicates. In this test, simulations are done by random sampling from the discrete distribution specified by the given theoretical distribution, each sample being of size *n* = sum(x), with x the numeric vector of absolute observed frequencies (see help of R for more details). To account for multiple testing, we used the *Benjamini–Hochberg* procedure to compute *q*-values.

## 3. RESULTS

### 3.1. Duplicated Genes in the Human Genome Are Mainly Located on Different Chromosomes, Represent Old Events and Display Similar TE Environment

Among the 10,064 homologous families present in the HOGENOM database grouping 16,144 human proteins, about 75% of the families contain single copy genes (7576 families). The 25% remaining families contain from 2 to 345 human genes. We decided to focus our analyses on gene families with two copies (that we will refer to in this manuscript as “duplicated genes”), which represent 14.53% of all gene families (1462 families containing 2924 proteins). Among the 2924 proteins, we were able to find the corresponding gene ids in Ensembl for 2840 genes.

**Figure 1:**
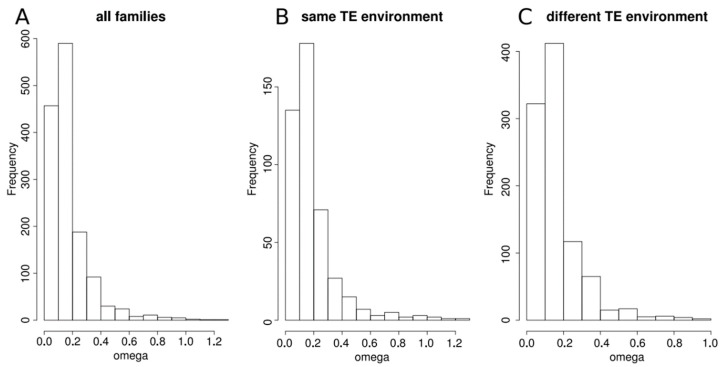
Distribution of the omega (dN/dS) ratio computed between duplicated genes from a same family, (**A**) for all families, (**B**) for families whose two genes have the same transposable elements (TE) environment, (**C**) for families whose two genes have different TE environment.

These duplicated genes are quite old as confirmed by the elevate mean synonymous rate we obtained when comparing the gene pairs (mean dS = 3.136). Indeed, this rate increases with the time since the duplication event [29]. Only 48 pairs of duplicated genes displayed dS values less than 0.25, which indicates that they represent very recent duplicates, among the 99 pairs of duplicated genes that we qualified as young families. Among the others, 189 pairs were considered as middle-age families and 1132 pairs were considered as old families. We determined the physical distances between the duplicated genes. The vast majority (2464 over 2840 genes representing 86.76% of all duplicated genes) is located on different chromosomes. Among the remaining 376, 26 duplicated genes are overlapping and the global distance between the other 350 duplicated genes is quite high since the median distance is about 81 kb (72kb when considering only young families and 109 kb when considering only middle-age and old families). When we looked at the position of genes according to the age of the family (young, middle-age and old), 72% and 94% of middle-age and old families, respectively, had their genes on different chromosomes, whereas only 37% of the young families had their genes on different chromosomes. We examined the level of sequence divergence by estimating the omega ratio (corresponding to the dN/dS ratio) for all duplicate pairs (Figure 1A). The ratios centered around a median at 0.129 with only four families with omega > 1. This indicates rather slow rates of protein evolution, suggesting that the genes of all these families are evolving under purifying selection.

We then explored the TE environment of the duplicated genes. All coding genes from the human genome were clustered according to their TE environment (see method) and we then considered only the duplicated genes. The distributions corresponding to the number of genes according to their TE neighborhood category between all genes in the genome and the duplicated genes are not different (Table 1; Χ-squared = 2.4439, df = 4, *p*-value = 0.6547).

In both cases, TE-free genes are the less abundant category since they represent less than 5% of all genes. The TE-very-rich genes are also less frequent (less than 14%). Both TE-very-poor and TE-rich genes represent the same proportion in the genome (>25%). The most represented category concerned the TE-poor genes (>30%). We then explored for each gene family if the two duplicated genes have similar TE environment. We observed that in a large proportion of the families (31.9%—453 families), the two genes are assigned to the same TE neighborhood cluster. This is significantly higher than when grouping randomly two genes (24%; Χ-squared = 35.584, df = 1, *p*-value = 2.443 × 10^−9^) and this remains significant when considering only families whose genes are located on different chromosomes. When considering all possible associations of TE context for duplicated gene pairs, their observed occurrences are significantly different than expected according to the frequencies of TE categories in the entire genome (Table 2; Χ-squared = 226.52, *p*-value estimated according to 2000 replicates using Monte Carlo test = 0.0004998).

Moreover, the results indicate that there is an excess of families whose genes are either in the same or in close categories (Table 2). This observation remains true when considering each class of age independently (Supplementary Table S1), although the comparison between the three age classes indicates that when the families are recent, the proportion of genes with the same TE environment is larger than in older families (Χ-squared = 8.65, df = 2, *p*-value = 0.01323). We looked at the omega ratio of gene families, taking into account the TE environment of their genes. For that, we separated families in which both genes had a similar TE environment and those in which genes had a different TE environment (Figure 1B,C). The distributions of the omega ratio are not different between the two groups (Two-sample Kolmogorov-Smirnov test D = 0.074374, *p*-value = 0.06847), indicating similar evolutionary constraints on the families, irrespective of their TE environment. This remains true whatever the age of the family.

### 3.2. Duplicated Genes Have Similar Histone Modification Enrichment Especially If They Share a Similar TE Environment

**Figure 2:**
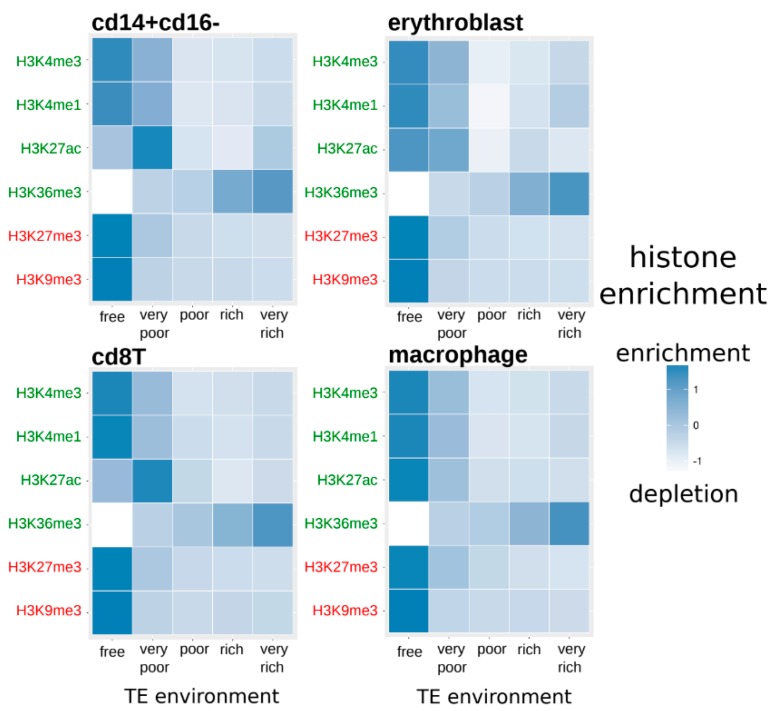
Normalized average histone enrichment of duplicated genes in each cell type according to the category related to their TE neighborhood (from TE-free to TE-very-rich genes). White color indicates a depletion in the considered histone modification and dark blue indicates an enrichment of the histone modification (Kruskal-Wallis tests, *q*-values < 0.05—Supplementary Table S2). Activating and repressive histone modifications are represented, respectively, in green and red.

We determined the histone enrichment of each duplicated gene according to their TE neighborhood in four cell types. Figure 2 displays the normalized average histone enrichment in each cell type and inside each gene category related to their TE neighborhood (from TE-free to TE-very-rich genes).

Inside each cell type and for each histone modification, there are significant differences between genes according to their TE neighborhood (Kruskal-Wallis tests, *q*-values < 0.05—Supplementary Table S2). In particular, for all histone modifications, there is a decrease in the histone enrichment of genes associated with an increase in the presence of TEs in their neighborhood, excepted for H3K36me3 for which it is the contrary with more enrichment when genes have a neighborhood richer in TEs.

We then wanted to determine if genes from the same family could have similar histone enrichment and if this could be linked with any similarity in the amount of TEs found nearby. We thus tested the correlations inside each family of the histone enrichment of genes (Table 3).

**Figure 3:**
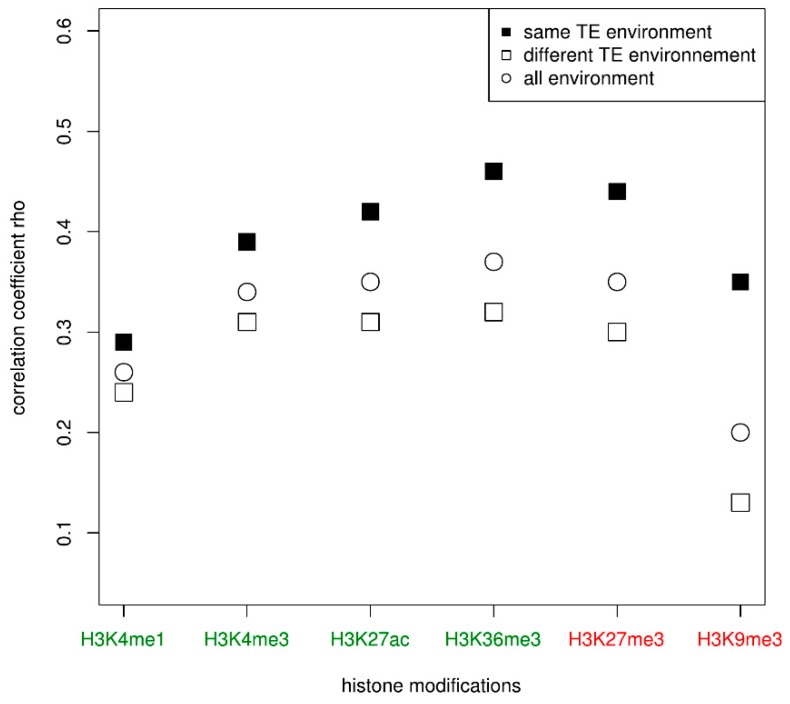
Correlations of the histone enrichment between paired genes according to the similarity of their TE neighborhood in the four cell types. Activating and repressive histone modifications are represented, respectively, in green and red.

The results showed an effect of the gene family since for all cell types and for all histone modifications, there are significant positive correlations between the histone enrichment of genes from the same family. According to the histone modification considered, the positive correlations are more or less pronounced. For example, the genes have a higher positive correlation for their enrichment in H3K27me3 (0.31 in CD14+CD16−, 0.34 in macrophages, 0.32 in CD8T and in erythroblasts) than in H3K9me3 (0.18 in CD14+CD16−, 0.17 in macrophages, 0.10 in CD8T, and 0.15 in erythroblasts). In order to determine if these positive correlations may be only due to the fact that genes are from the same gene family or if their respective TE environment may be involved, we tested the same correlations between duplicated genes having similar TE environment on one hand and between duplicated genes with different TE environment on the other hand. The second case is expected to underline any correlations due only to the belonging of the same family. In that last case, we observed positive correlations but they were weaker than when considering all genes (Table 3). For the histone modification H3K9me3, the correlations even disappeared in CD8T and was barely significant in erythroblasts. However, when considering only gene families for which both genes have similar TE environment, the positive correlations observed before were stronger, especially for the H3K27me3 modification (0.41 in CD14+CD16−, 0.40 in macrophages, 0.43 in CD8T and 0.44 in erythroblasts). In Figure 3 (Supplementary Table S3), we displayed the correlations for each histone modification and according to the TE neighborhood, for all cell types taken together.

The figure clearly shows that genes of a given family that display a similar TE environment have a stronger positive correlation for their histone modification enrichment compared to genes that have a different TE environment. For example, the Spearman correlation rho of the duplicated genes for their enrichment in H3K36me3 is 0.37 when it increases to 0.46 when considering only families whose duplicates share the same TE environment (Supplementary Table S3). To determine whether this correlation may be linked to the age of the family, we computed the correlations in the three age groups. The previous observation remains globally true in some cell types, especially for middle-age families (Supplementary Table S4). Indeed, irrespective of the cell type and the histone modification, duplicated genes having the same TE environment usually displayed a positive correlation for their histone enrichment that is stronger than duplicated genes with different TE environment when they belong to middle-age families. For the histone modifications H3K36me3 and H3K27me3, this positive correlation is also observed between genes from young families. However, the correlation is generally less strong or at least the same between genes from old families, independently of the TE environment with few exceptions. For example, there is a higher positive correlation for H3K36me3 enrichment in macrophage and erythroblast among duplicated genes with the same TE environment in old families when compared to genes with a different TE environment, which is not the case in CD14+CD16 and CD8T in which the positive correlation is the same irrespective of the TE environment.

### 3.3. Duplicated Genes Have a Similar Methylation Level That Is Linked to Both the TE Environment Conservation and the Age of the Gene Family

**Figure 4:**
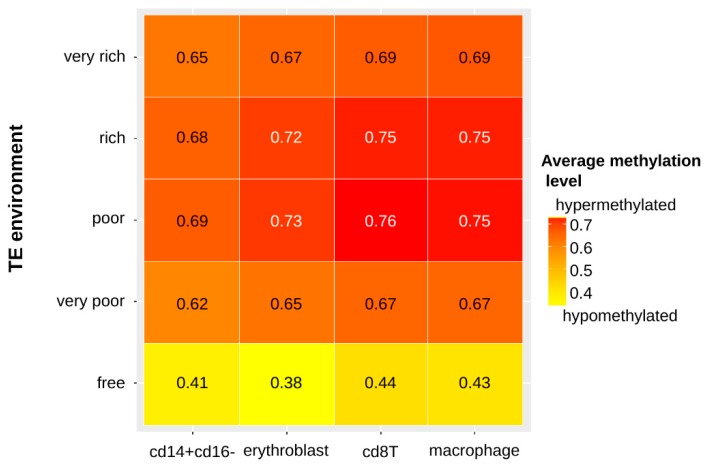
Average methylation level of the duplicated genes according to their neighborhood in TEs in the four cell types. (Kruskal-Wallis tests, *p*-values < 2.2 × 10^−16^).

We looked at the methylation level of each duplicated gene linked with its richness in TEs in the four cell types (Figure 4).

In all cell types, the methylation level of the genes is associated with the TE category of each gene (Kruskal-Wallis tests, *p*-values < 2.2 × 10^−16^). In particular, TE-free genes are systematically less methylated than the other genes. Interestingly, the genes categorized as TE-poor and TE-rich displayed the highest methylation levels when we could have expected TE-very-rich genes to behave this way if the presence of TEs was mainly responsible for the methylation level of the genes.

We then compared inside each gene family the methylation level of the duplicated genes (Table 4).

We observed that there is a positive correlation in the methylation level between the genes belonging to the same family. For example, in the macrophage cell type, the Spearman correlation rho is 0.18 (*q* value = 6.97 × 10^−11^), indicating a weak but significant positive correlation. To investigate the implication of the quantity of nearby TEs around the genes, we compared the genes only from families whose two genes had similar TE environment. In that case, we observed a stronger positive correlation (Table 4). For example, in the macrophage cell type, the Spearman rho is 0.27 (*q* value = 1.33 × 10^−8^). On the contrary, when considering families in which the two genes have a different TE environment, although there is still a positive correlation, it is weaker (for example, r = 0.13, *q* value = 4.40 × 10^−5^ in the macrophage cell type).

We investigated whether the age of the family may be implicated in the observed correlations. There is a positive correlation in the methylation level between the duplicated genes for young and middle age families but this correlation is either absent or not significant for old families (Supplementary Table S5). When taking into account the TE environment around the genes, the conservation of the methylation level is higher in young and middle-age families for families whose genes have a similar TE environment, except for young families with respect to erythroblasts (Supplementary Table S5). This is true also for old families for three cell types (erythroblast, macrophage, and CD8T), although the correlation values are weak. It thus seems that in the case of methylation conservation, the age of the family plays an important role, in addition to the TE environment.

### 3.4. The Duplicated Genes with Very Low Expression Divergence Display a High Conservation of Epigenetic Modifications and TE Environment

We found that the majority of the genes are either expressed in the four cell types (*n* = 1677—59.05%) or in none of them (*n* = 729—25.67%). Of the 1420 families, genes from 267 of them presented no expression in all cell types for both members of the pair. These gene families were not considered in the remaining analyses.

**Figure 5:**
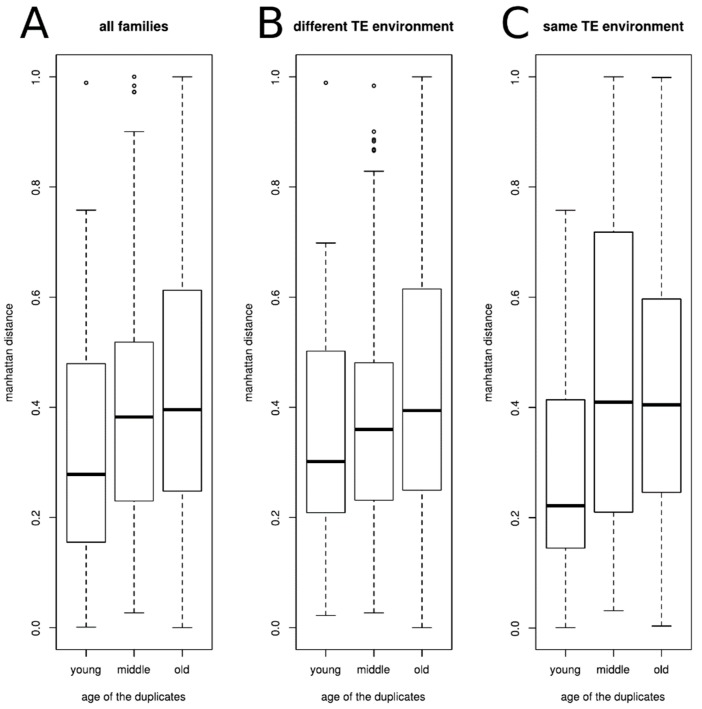
Average Manhattan distance of the duplicated gene expression level inside each class of ages, (**A**) for all families (Kruskal-Wallis chi-squared = 15.789, df = 2, *p*-value = 0.0003727), (**B**) for families whose two genes have different TE environment (Kruskal-Wallis chi-squared = 7.8622, df = 2, *p*-value = 0.01962), (**C**) for families whose two genes have the same TE environment (Kruskal-Wallis chi-squared = 10.421, df = 2, *p*-value = 0.00546).

To determine the divergence in expression between duplicates of a given family, we computed the normalized Manhattan distance d_m_ to compare differences in the relative abundance of the two genes across the four cell types. We looked at the average Manhattan distance inside each class of ages (Figure 5A).

As could be expected, the divergence of expression between the genes is associated with the age of the family, genes from old families having an expression divergence higher than genes from young families (Kruskal-Wallis chi-squared = 15.789, df = 2, *p*-value = 0.0003727). We then tested whether the TE environment around the genes could be associated with the observed expression divergence. For that, we separated the gene families according to the TE environment of the duplicated genes (similar or different). The results are presented on the Figure 5B,C. We observed the same tendency as when all gene families were considered together, irrespective of the TE environment, to have a difference in gene expression divergence according to the age of the families (different TE environment, Kruskal-Wallis chi-squared = 7.8622, df = 2, *p*-value = 0.01962; same TE environment Kruskal-Wallis chi-squared = 10.421, df = 2, *p*-value = 0.00546). However, the expression divergence is the same for a given class of age independently of the TE environment (Two-sample Kolmogorov-Smirnov tests D = 0.29895, *p*-value = 0.1048, D = 0.21009, *p*-value = 0.1433, and D = 0.03746, *p*-value = 0.9661 for young, middle-age, and old families, respectively).

We then studied the association between the different types of epigenetic modifications of the duplicated genes, the expression divergence and the TE environment. We separated the gene families according to the level of expression divergence in four classes (very low d_m_ [0–0.25], low d_m_ [0.25–0.5], medium d_m_ [0.5–0.75], and high d_m_ [0.75–1]). The results showed that a strong positive correlation in the DNA methylation level can be observed only for the families whose duplicates share similar TE environment and have a very low expression divergence (Spearman correlation rho = 0.33, *q* value = 6.88 × 10^−3^; Supplementary Table S6). We performed the same kind of analysis considering the mean histone modification enrichment between the duplicated genes of each family (Supplementary Table S6). As previously, we separated the gene families according to the level of gene expression divergence. The results showed also a strong positive correlation of the histone enrichment between paired genes when they have similar TE environment and a very low expression divergence (from rho = 0.31 *q* value = 1.28 × 10^−2^ for H3K9me3 to rho = 0.57, *q* value = 1.77 × 10^−7^ for H3K4me3 Supplementary Table S6). A less strong positive correlation is also observed for three histone modifications (H3K4me3, H3K36me3 and H3K9me3) for genes with a low expression divergence and a same TE environment (rho = 0.29 *q* value = 8.16 × 10^−3^, rho = 0.29 *q* value = 8.16 × 10^−3^, and rho = 0.26 *q* value = 2.09 × 10^−2^, respectively). There is also a positive correlation for families whose genes have a different TE environment and a very low expression divergence for H3K4me3, H3K27ac, H3K36me3, and H3K27me3, these correlations being less strong than for genes having the same TE environment (rho = 0.33 *q* value = 5.23 × 10^−5^, rho = 0.29 *q* value = 4.65 × 10^−4^, rho = 0.25 *q* value = 3.16 × 10^−3^, and rho = 0.19 *q* value = 3.10 × 10^−2^, respectively). The conservation of epigenetic modifications and of the TE environment around genes is associated with a very low expression divergence between duplicated genes.

## 4. Discussion

The maintenance and evolution of duplicated genes have been proposed to be linked to variation in epigenetic modifications [65]. For example, it has been shown in zebrafish that epigenetic divergence of duplicated genes affects both their expression and their functional divergence [66]. In humans, duplicated genes display highly consistent patterns of DNA methylation divergence across multiple tissues due to different frequencies of sequence motifs, which allowed the proposal that DNA methylation could be a causative factor for functional divergence of duplicated genes [29]. In *Arabidopsis*, the presence of H3K27me3 correlates with a slower rate of function evolution in duplicated gene families [30]. These various examples indicate that epigenetic modifications can have an evolutionary importance in the fate of duplicated genes. To gain insight into this question, we have analyzed in this work different histone modifications enrichment and DNA methylation level between pairs of genes from a same family, in different cell types, taking into account the presence of TEs near the genes, to evaluate their impact in any potential conservation or divergence of these epigenetic modifications.

We have focused our interest on gene families of size two. They represent the majority of the multigenic families in the human genome. We have observed that on average, these families are quite old. This is consistent with the hypothesis of the two rounds of whole genome duplication that occurred early in the evolution of vertebrates [67,68]. It was indeed predicted that we should have an excess of two and four size gene families in the human genome due to extensive gene losses that occurred later [69]. Even if the average age is quite high for those families, there remains a substantial number of families that appeared recently, via other mechanisms. Their synonymous substitution rate distribution is consistent with what was observed for all duplicated genes with Ks values less than 1 [70], indicating that the young families of size two are representative of all young duplicated families. When we analyzed the TE environment around these duplicated genes, we found that the proportion of TEs around them is not different than when considering all human genes, with TE-free genes being the less abundant category of genes, followed by TE-rich genes. Interestingly, we observed that genes from the same family tend to globally conserve the same type of TE neighborhood. It could be expected to observe this tendency only for young gene families, whose genes did not have time to differently accumulate new TE insertions. Young families indeed present an excess of similar TE environments in both genes. However, it is also true for older gene families, even if the proportion decreases. Although this indicates a link between the conservation of TE neighborhood between duplicated genes and the age of the duplication, it is not the only explanation since we can still observe this effect in old families. In old families, we could also hypothesize that some selective pressures to conserve the gene environment are at work that could explain the similar TE environment. The duplicated genes displayed a similar level of selection acting on them, indicating that almost all genes in our dataset evolve under purifying selection. These selective pressures could thus explain why genes from the same family tend to conserve the same TE environment. Selective constraints acting on genes have already been shown to be associated with the presence of TE insertions near the genes [71,72]. In particular, TE-free genes were shown to be subjected to a stronger purifying selection when compared to TE-rich genes [72]. However, the same selective pressure is also acting on gene pairs for which the TE environment is different. Then, the purifying selection that could act against TE insertions is not enough to explain why the members of some gene families conserve the same TE environment. Another possibility to explain the conservation of the TE environment could be linked with the gene function of duplicated genes. However, we did not detect any functional bias among the duplicated genes with the same TE environment when compared to all duplicated genes in the human genome. To go deeper to explore this question would be to focus more specifically on larger gene families for which more data are available concerning their function.

We have shown in this work that according to the proportion of TEs inserted near genes, there are variations in the level of methylation and the enrichment in histone modification of genes. In particular, TE-free genes are depleted in H3K36me3 whereas TE-very-rich genes are on the contrary enriched for this modification. This modification has usually been described to be associated with active chromatin but it has also been shown to be implicated in various other mechanisms like transcriptional repression, alternative splicing or DNA methylation [73]. Interestingly, this modification can promote repressive chromatin within actively transcribed genes, preventing spurious transcription initiation from cryptic promoters or TE remnants [74]. The histone modifications H3K4me1, H3K4me3, H3K27ac, H3K27me3 and H3K9me3 were found to be more present in TE-free genes rather than in genes with TEs in their surroundings. This could be expected for the modifications H3K4me1, H3K4me3, and H3K27ac, which have been shown to be associated with actively transcribed regions, if we consider that TEs are rather associated with repressive modifications [63,75]. This could be more surprising concerning the repressive modifications H3K27me3 and H3K9me3, which have been shown to be associated with TE repression in various cell types and organisms [43,63,75,76,77,78]. Since histone modifications can spread at TE insertions [46], it could be expected that genomic regions with numerous TE insertions would be impacted by repressive modifications originating in TEs. However, in this work, we are considering TEs that are found near or in genes, rather than intergenic insertions. We are thus considering TE insertions among which some could potentially have a role in the regulation of gene expression and some could just be neutral with no particular effect. Indeed, it has been observed that SINE elements are depleted in H3K9me3, especially when they are close to genes, supporting a potential role of these elements in the gene regulation [76]. Moreover, we already observed these results in other work [53], that could be explained by the “exaptation hypothesis” [77], considering that epigenetic modifications associated with specific TE insertions could be adaptive. This would imply that among all TE insertions in a genome, not all of them will have the same impact on gene expression. We also observed that TE-free genes displayed the lowest level of methylation when compared to genes with TEs in their surrounding. This is what could be expected if the presence of TEs in or near genes triggers DNA methylation, since this epigenetic modification has been largely associated with TE silencing, especially in mammals [79]. Interestingly, the proportion of TEs does not seem to impact the level of methylation since even TE-very-poor genes displayed as much DNA methylation than TE-very-rich genes. This could indicate that the methylation level does not increase with the number of TEs but as soon as even a few TEs are present, they are susceptible to trigger a significant amount of methylation.

We compared the histone enrichment and methylation level between both members of the same gene family in four different cell types to determine whether duplicated genes tend to conserve their epigenetic environment. As we could expect, there is a positive correlation of the epigenetic modification between genes from the same family, especially when the families are young. This is consistent with what was previously observed concerning the DNA methylation divergence of duplicated genes, with young duplicates displaying similar levels of methylation compared to older duplicates [29,31]. This could be explained by the fact that young duplicates are likely to be in a similar genomic environment. Indeed, when we considered only young duplicates (99 pairs of genes), there is a strong positive correlation for the histone enrichment irrespective of the TE environment, when the two genes are on the same chromosome (62 pairs of genes) (Supplementary Table S7). However, the duplicated genes we analyzed are on average very far away and sometimes even on different chromosomes. The conservation of epigenetic modification is in contradiction to the results presented by a study on segmental duplications in which an asymmetry was observed in the methylation level and in the histone acetylation that could be linked to pseudogenization [80]. Although in this last study, the genes considered may not all be pseudogenes, the discrepancy could be explained by the fact that in our work, we focused only on duplicated genes that are both functional. It was proposed that when a gene is in a different genomic environment, this could trigger changes in epigenetic modifications that could allow new duplicates to be submitted to new selective pressures preventing their pseudogenization [81]. The correlation we observed is stronger when the TE environment of both genes is similar. This could be a byproduct of selective pressure acting on those genes that would have the consequence to conserve the same proportion of TE insertions by removing any new insertions. However, when duplicated genes with different TE environments are submitted to the same selective constraints, then the selective pressure acting on duplicated genes is not enough to explain this observation. In *Arabidopsis*, there is an association between the conservation of H36K27me3 of paralogs and conserved noncoding sequences (CNS) [82]. The same mechanism could be at work in this case. However, we did not find much overlap between the duplicated genes and CNS previously identified in humans [83]. Only 17% (*n* = 484) of the duplicated genes from our analysis were overlapping with at least one CNS. This overlapping concerned the two duplicated genes of only 49 gene families. The presence of TEs could thus be implicated in both the maintenance and the divergence of epigenetic modifications.

In conclusion, our results point out the possibility for TE insertions to participate in the modulation of epigenetic variation of genes, especially inside duplicated gene families. New TE insertions could help trigger new epigenetic modifications that could have an impact in the functional divergence of the duplicated genes, whereas ancestral insertions would on the contrary have an effect of conservation. This hypothesis is supported by the fact that we observed a strong positive correlation in epigenetic modification between both duplicates when they display very low expression divergence and the same TE environment, irrespective of the age of the family. Perspectives on this work will require to work at the individual TE insertion level in order to identify, without any ambiguity, epigenetic modifications associated with them to clearly identify their effect on gene regulation.

## Figures and Tables

**Table 1:** Number of genes according to their TE neighborhood category.

TE Category	All Protein Coding Genes	Duplicated Genes
TE-free	773 (4.08%)	109 (3.84%)
TE-very-poor	4830 (25.50%)	713 (25.10%)
TE-poor	5885 (31.08%)	915 (32.22%)
TE-rich	4848 (25.60%)	729 (25.67%)
TE-very-rich	2602 (13.74%)	374 (13.17%)

**Table 2:** Number of gene families according to the TE neighborhood category of each duplicated gene.

		TE Environment of the Second Gene				
		TE-Free	TE-Very-Poor	TE-Poor	TE-Rich	TE-Very-Rich
**TE Environment of the First Gene**	TE-free	**20 (1.41%)**	/	/	/	/
	TE-very-poor	**36 (2.53%)**	**121 (8.52%)**	/	/	/
	TE-poor	*18 **(1.27%)***	220 **(15.49%)**	**169 (11.90%)**	/	/
	TE-rich	*13 **(0.91%)***	*143 **(10.07%)***	229 **(16.13%)**	**110 (7.75%)**	/
	TE-very-rich	*2 **(0.14%)***	*72 **(5.07%)***	*110 **(7.75%)***	**124 (8.73%)**	**33 (2.321%)**

In bold: excess; italic: depletion; the percentages of gene families are indicated in parenthesis.

**Table 3:** Correlations of the histone enrichment between paired genes.

		Repressive Modifications				Activating Modifications							
		H3K27me3		H3K9me3		H3K36me3		H3K27ac		H3K4me1		H3K4me3	
		Spearman Rho	*q* Value	Spearman Rho	*q* Value	Spearman Rho	*q* Value	Spearman Rho	*q* Value	Spearman Rho	*q* Value	Spearman Rho	*q* Value
Duplicated genes	CD14+CD16−	0.31 *	4.525714 × 10^−16^	0.18 *	2.767059 × 10^−11^	0.29 *	4.525714 × 10^−16^	0.31 *	4.525714 × 10^−16^	0.19 *	1.496681 × 10^−13^	0.33 *	4.525714 × 10^−16^
	erythroblast	0.32 *	4.525714 × 10^−16^	0.15 *	2.088000 × 10^−8^	0.30 *	4.525714 × 10^−16^	0.31 *	4.525714 × 10^−16^	0.27 *	4.525714 × 10^−16^	0.39 *	4.525714 × 10^−16^
	CD8T	0.32 *	4.525714 × 10^−16^	0.10 *	1.878261 × 10^−4^	0.29 *	4.525714 × 10^−16^	0.16 *	4.189091 × 10^−9^	0.24 *	4.525714 × 10^−16^	0.30 *	4.525714 × 10^−16^
	macrophage	0.34 *	4.525714 × 10^−16^	0.17 *	1.369385 × 10^−10^	0.30 *	4.525714 × 10^−16^	0.23 *	4.525714 × 10^−16^	0.20 *	1.224000 × 10^−14^	0.33 *	4.525714 × 10^−16^
Duplicated genes with same TE environment	CD14+CD16−	0.41 *	4.525714 × 10^−16^	0.25 *	4.865806 × 10^−8^	0.36 *	8.623256 × 10^−15^	0.38 *	4.525714 × 10^−16^	0.21 *	7.111385 × 10^−6^	0.35 *	1.936000 × 10^−14^
	erythroblast	0.44 *	4.525714 × 10^−16^	0.29 *	4.727547 × 10^−10^	0.38 *	4.525714 × 10^−16^	0.38 *	4.525714 × 10^−16^	0.27 *	4.189091 × 10^−9^	0.42 *	4.525714 × 10^−16^
	CD8T	0.43 *	4.525714 × 10^−16^	0.22 *	1.714286 × 10^−6^	0.32 *	4.838400 × 10^−12^	0.26 *	3.234098 × 10^−8^	0.26 *	2.088000 × 10^−8^	0.36 *	5.356098 × 10^−15^
	macrophage	0.40 *	4.525714 × 10^−16^	0.26 *	1.743158 × 10^−8^	0.44 *	4.525714 × 10^−16^	0.19 *	3.299104 × 10^−5^	0.22 *	1.890000 × 10^−6^	0.36 *	5.356098 × 10^−15^
Duplicated genes with different TE environment	CD14+CD16−	0.25 *	2.368421 × 10^−15^	0.14 *	1.963636 × 10^−5^	0.25 *	7.645714 × 10^−15^	0.27 *	4.525714 × 10^−16^	0.18 *	1.998621 × 10^−8^	0.31 *	4.525714 × 10^−16^
	erythroblast	0.26 *	7.297297 × 10^−16^	0.08 *	1.454197 × 10^−2^	0.26 *	4.525714 × 10^−16^	0.27 *	4.525714 × 10^−16^	0.26 *	4.940000 × 10^−16^	0.36 *	4.525714 × 10^−16^
	CD8T	0.25 *	4.098462 × 10^−15^	0.03	2.789000 × 10^−1^	0.28 *	4.525714 × 10^−16^	0.11 *	5.142857 × 10^−4^	0.22 *	4.599184 × 10^−12^	0.26 *	4.525714 × 10^−16^
	macrophage	0.31 *	4.525714 × 10^−16^	0.12 *	1.058824 × 10^−4^	0.22 *	3.750000 × 10^−12^	0.24 *	8.405217 × 10^−14^	0.18 *	7.315714 × 10^−9^	0.31 *	4.525714 × 10^−16^

bold * statistically significant correlations (*q* values < 0.05).

**Table 4:** Correlations of the methylation level between duplicated genes.

	Duplicated Genes		Duplicated Genes with the Same TE Environment		Duplicated Genes with a Different TE Environment	
	Spearman Rho	*q* Value	Spearman Rho	*q* Value	Spearman Rho	*q* Value
CD14+CD16−	0.14 *	1.911360 × 10^−6^	0.17 *	3.354545 × 10^−4^	0.11 *	9.217000 × 10^−4^
erythroblast	0.16 *	9.492000 × 10^−9^	0.21 *	2.280000 × 10^−5^	0.13 *	3.475500 × 10^−5^
CD8T	0.15 *	1.333800 × 10^−8^	0.22 *	9.620000 × 10^−6^	0.12 *	1.370400 × 10^−4^
macrophage	0.18 *	6.968400 × 10^−11^	0.27 *	1.333800 × 10^−8^	0.13 *	4.400000× 10^−5^

bold * statistically significant correlations (*q* values < 0.05).

## Supplementary Materials

The following are available online at https://www.mdpi.com/2073-4425/10/3/249/s1. Supplementary data S1: List and positions of the duplicated genes used in this work. Supplementary Table S1: Number of gene families according to the TE neighborhood category of each duplicated gene and the age of the families. Supplementary Table S2: mean histone enrichment of genes for each tissue type and according to their TE neighborhood. Supplementary Table S3: correlations of histone enrichment between duplicated genes of each family across all cell types. Supplementary Table S4: correlation of the histone enrichment between genes from the same family, according to the TE neighborhood and the age of the family. Supplementary Table S5: correlations of the methylation level between duplicated genes from a same family, according to the age of the family and the TE neighborhood. Supplementary Table S6: correlations of the methylation level or histone enrichment of the duplicated genes according to the level of expression divergence between the two genes across all tissues. Supplementary Table S7: correlation of the histone enrichment between genes from the same young family, according to the TE neighborhood and the position on the chromosome.
