# Supplementary material for: Does the Presence of Transposable Elements Impact the Epigenetic Environment of Human Duplicated Genes?
Source: Genes (Basel). 2019 Mar 26;10(3):249. doi: 10.3390/genes10030249 (PMC6470583; doi:10.3390/genes10030249)
Supplement: Supplementary file 1 [file genes-10-00249-s001.pdf]

Supplementary Table S1: Number of gene families according to the TE neighborhood category of each duplicated gene and the age of the families – Statistical tests (chi-squared tests for given probabilities with simulated p-values based on 2,000 replicates) made by comparison to all duplicated families.

		**TE free**	**TE very poor**	**TE poor**	**TE rich**	**TE very rich**
**Old families**	**TE free**	**17**	/	/	/	/
	**TE very poor**	**28**	**93**	/	/	/
	**TE poor**	*14*	*182*	**133**	/	/
	**TE rich**	*10*	*119*	**191**	**77**	/
	**TE very rich**	*2*	*60*	*92*	**86**	**23**
	X-squared = 177.29, df= NA, *p*-value = 0.0004998					
**Middle-age families**	**TE free**	**2**	/	/	/	/
	**TE very poor**	*1*	**16**	/	/	/
	**TE poor**	**3**	*23*	**24**	/	/
	**TE rich**	*2*	*19*	*25*	**20**	/
	**TE very rich**	*0*	*7*	*14*	**28**	**5**
	X-squared = 51.141, df= NA, *p*-value = 0.001499					
**Young families**	**TE free**	**1**	/	/	/	/
	**TE very poor**	**7**	**12**	/	/	/
	**TE poor**	*1*	*12*	**12**	/	/
	**TE rich**	*1*	*5*	*13*	**13**	/
	**TE very rich**	*0*	*4*	*2*	**10**	**5**
	X-squared = 41.184, df= NA, *p*-value = 0.002999					

In bold: excess; italic: depletion

Supplementary Table S2: Mean histone enrichment of genes for each tissue type and according their TE neighborhood

Cell types	Histone modifications	TE-free	TE- -poor	TE-poor	TE-rich	TE-very-rich	*Kruskal-Wallis chi-squared*	*q values*
cd14+cd16-	H3K4me3	6.807534	5.133605	3.030559	3.091142	3.425793	*31.735*	2.476571×10^{-6}
	H3K4me1	0.9012089	0.7732764	0.5453501	0.5541241	0.6000732	*26.1*	3.022000×10^{-5}
	H3K27ac	2.792989	3.34889	2.500225	2.449032	2.735089	*61.215*	3.514909×10^{-12}
	H3K36me3	0.1760073	0.2505129	0.2561121	0.320778	0.3527147	*116.54*	6.600000×10^{-16}
	H3K27me3	0.77653299	0.24382813	0.12426352	0.08844069	0.0735982	*200*	6.600000×10^{-16}
	H3K9me3	0.39785677	0.06098398	0.0305218	0.02869545	0.02065048	*60.094*	5.544000×10^{-12}
erythroblast	H3K4me3	1.9410196	1.5390392	0.9861545	1.0727333	1.2006346	*45.497*	5.008000×10^{-9}
	H3K4me1	0.6813305	0.5595872	0.4252929	0.4737135	0.5219408	*31.503*	2.640000×10^{-6}
	H3K27ac	1.5271913	1.4116306	0.8968728	1.0609551	0.9632104	*69.793*	6.024000×10^{-14}
	H3K36me3	0.1929647	0.2586508	0.2757881	0.3415434	0.3906885	*134.13*	6.600000×10^{-16}
	H3K27me3	0.91705601	0.25656121	0.11908253	0.07724129	0.06472285	*213.11*	6.600000×10^{-16}
	H3K9me3	0.32597173	0.06011478	0.04114728	0.03717132	0.0348549	*54.295*	7.834286×10^{-11}
cd8T	H3K4me3	4.84842	3.069301	1.841949	1.89959	2.043597	*36.134*	3.435789×10^{-7}
	H3K4me1	0.8692454	0.560045	0.3930538	0.3720045	0.4040655	*96.415*	6.600000×10^{-16}
	H3K27ac	0.227859	0.2860348	0.1966394	0.1781869	0.1916664	*39.801*	6.720000×10^{-8}
	H3K36me3	0.1189102	0.2166163	0.2401037	0.2801871	0.3463692	*126.43*	6.600000×10^{-16}
	H3K27me3	0.50704555	0.14168205	0.05477228	0.03841184	0.03079635	*217.57*	6.600000×10^{-16}
	H3K9me3	0.29469777	0.04096908	0.02032979	0.030825	0.02855964	*40.274*	5.700000×10^{-8}
macrophage	H3K4me3	2.3838373	1.5027608	0.8866685	0.9413567	1.0405502	*30.735*	3.620870×10^{-6}
	H3K4me1	0.6230498	0.4874439	0.3879674	0.3939001	0.4210371	*38.307*	1.292000×10^{-7}
	H3K27ac	1.0389236	0.5900725	0.3557303	0.3638086	0.359079	*33.596*	1.082400×10^{-6}
	H3K36me3	0.1831851	0.2409833	0.246001	0.27591	0.3207072	*74.434*	7.013333×10^{-15}
	H3K27me3	0.49491689	0.21780921	0.12708917	0.08795114	0.06863786	*181.99*	6.600000×10^{-16}
	H3K9me3	0.34171069	0.06738416	0.05208118	0.05379619	0.0463803	*57.067*	2.215385×10^{-11}

In green are represented activating histone modifications and in red the repressing histone modifications.

Supplementary table S3: Correlations of histone enrichment between duplicated genes of each family across all cell types

	All TE environment		Same TE environment		Different TE environment	
	**Rho**	***q* value**	**Rho**	***q* value**	**Rho**	***q* value**
H3K4me1	**0.26***	3.046154×10^{-16}	**0.29***	1.551176×10^{-10}	**0.24***	1.402875×10^{-14}
H3K4me3	**0.34***	3.046154×10^{-16}	**0.39***	3.046154×10^{-16}	**0.31**	3.046154×10^{-16}
H3K27ac	**0.35***	3.046154×10^{-16}	**0.42***	3.046154×10^{-16}	**0.31**	3.046154×10^{-16}
H3K36me3	**0.37***	3.046154×10^{-16}	**0.46***	3.046154×10^{-16}	**0.32***	3.046154×10^{-16}
H3K27me3	**0.35***	3.046154×10^{-16}	**0.44***	3.046154×10^{-16}	**0.30***	3.046154×10^{-16}
H3K9me3	**0.20***	7.602429×10^{-15}	**0.35***	1.357200×10^{-14}	**0.13***	3.635000×10^{-5}

*statistically significant correlations (q values < 0.05). In green are represented activating histone modifications and in red the repressing histone modifications.

Supplementary Table S4: Correlation of the histone enrichment between genes from a same family, according to the TE neighborhood and the age of the family

			Macrophage			Erythroblast			cd14+cd16-			cd8T		
			**young**	**middle**	**old**	**young**	**middle**	**old**	**young**	**middle**	**old**	**young**	**middle**	**old**
H3K36me3	Same TE environment	rho	**0.73***	**0.52***	**0.36***	**0.69***	**0.33***	**0.33***	**0.59***	**0.48***	**0.29***	**0.43***	**0.38***	**0.29***
		q value	8.059449×10^{-8}	1.746228×10^{-5}	1.773865×10^{-11}	1.034691×10^{-6}	0.007847694	1.56784×10^{-9}	5.388883×10^{-5}	6.714959×10^{-5}	2.027128×10^{-7}	0.005589463	0.002253915	2.307166×10^{-7}
	Different TE environment	rho	**0.33***	**0.25***	**0.19***	**0.31***	**0.29***	**0.25***	0.26	**0.30***	**0.23***	0.26	**0.20***	**0.29***
		q value	0.01549659	0.007130352	1.49947×10^{-7}	0.02689986	0.001594836	8.682229×10^{-12}	0.06408444	0.001339232	1.662856×10^{-10}	0.05835342	0.03589282	2.324155×10^{-15}
	All TE environment	rho	**0.48***	**0.36***	**0.25***	**0.45***	**0.33***	**0.27***	**0.39***	**0.39***	**0.25***	**0.32***	**0.28***	**0.29***
		q value	1.194391×10^{-6}	1.225144×10^{-6}	1.602788×10^{-16}	6.944531×10^{-6}	7.732592×10^{-6}	8.681842×10^{-20}	0.0001084915	1.196143×10^{-7}	1.16647×10^{-16}	0.001725068	0.0002120468	2.085925×10^{-21}
H3K27me3	Same TE environment	rho	**0.48***	**0.32***	**0.39***	**0.49***	**0.29***	**0.43***	**0.74***	**0.30***	**0.38***	**0.49***	**0.36***	**0.42***
		q value	0.001926112	0.01069249	3.001812×10^{-13}	0.001483524	0.02253684	3.105912×10^{-16}	7.563639×10^{-8}	0.01789455	2.033475×10^{-12}	0.001531875	0.003694703	2.037872×10^{-15}
	Different TE environment	rho	-0.05	**0.31***	**0.32***	0.24	0.03	**0.28***	**0.45***	0.15	**0.25***	-0.11	0.14	**0.29***
		q value	0.7192899	0.0007169657	1.531727×10^{-18}	0.08455906	0.741512	7.47716×10^{-15}	0.0008712583	0.1049185	7.799822×10^{-12}	0.4443859	0.1339991	1.781286×10^{-13}
	All TE environment	rho	0.15	**0.33***	**0.34***	**0.35***	0.12	**0.33***	**0.56***	**0.22***	**0.29***	0.13	**0.24**	**0.32***
		q value	0.1433032	9.714665×10^{-6}	2.538323×10^{-30}	0.0005727606	0.1247961	1.317086×10^{-28}	7.10407×10^{-9}	0.003559235	1.237251×10^{-22}	0.2026198	0.001737122	2.793755×10^{-27}
H3K27ac	Same TE environment	rho	0.21	**0.26***	**0.16***	0.26	**0.51***	**0.35***	**0.36***	**0.51***	**0.33***	**0.36***	0.22	**0.25***
		q value	0.1909829	0.03589282	0.00547132	0.102047	2.418087×10^{-5}	8.681675×10^{-11}	0.02053491	1.978458×10^{-5}	1.142405×10^{-9}	0.02342728	0.07975529	7.732592×10^{-6}
	Different TE environment	rho	**0.32***	0.14	**0.24***	**0.49***	**0.20***	**0.26***	**0.47***	**0.29***	**0.25***	0.13	0.16	**0.098***
		q value	0.02178147	0.1323728	3.252629×10^{-11}	0.0002018531	0.03124962	3.001812×10^{-13}	0.0005154156	0.00207511	7.799822×10^{-12}	0.3683611	0.08778437	0.008155402
	All TE environment	rho	**0.29***	**0.19***	**0.22***	**0.41***	**0.33***	**0.30***	**0.43***	**0.40***	**0.28***	**0.23***	**0.18***	**0.14***
		q value	0.005432234	0.01239393	4.458993×10^{-13}	5.974312×10^{-5}	9.667653×10^{-6}	7.904797×10^{-23}	2.23525×10^{-5}	6.453961×10^{-8}	1.859568×10^{-20}	0.02931879	0.01677197	4.433183×10^{-6}
H3K9me3	Same TE environment	rho	0.19	**0.42***	**0.22***	**0.53***	**0.35***	**0.25***	0.076	**0.25***	**0.26***	0.20	**0.36***	**0.19***
		q value	0.2411337	0.0006520568	6.652933×10^{-5}	0.0004280106	0.005753973	5.05526×10^{-6}	0.6415775	0.04485224	3.079152×10^{-6}	0.2179145	0.003559235	0.0008680394
	Different TE environment	rho	0.16	0.14	**0.11***	-0.01	0.11	**0.08***	0.02	0.18	**0.14***	-0.02	0.07	0.03
		q value	0.2411337	0.1304653	0.001839857	0.9122985	0.2569611	0.03278351	0.8666344	0.0524843	0.0002055909	0.8666344	0.4443859	0.4443859
	All TE environment	rho	0.18	**0.26***	**0.15***	0.16	**0.20***	**0.13***	0.05	**0.21***	**0.18***	0.06	**0.20***	**0.08***
		q value	0.08617591	0.0006217339	1.194391×10^{-6}	0.1208111	0.008691221	1.206248×10^{-5}	0.6385059	0.005480025	0.000000008	0.5794068	0.008028533	0.009428178
H3K4me3	Same TE environment	rho	**0.57***	**0.55***	**0.26***	**0.58***	**0.53***	**0.37***	**0.54***	**0.58***	**0.24***	**0.46***	**0.58***	**0.27***
		q value	0.0001487137	3.473284×10^{-6}	2.025632×10^{-6}	0.0001062559	1.208111×10^{-5}	1.522646×10^{-11}	0.0003626928	0.000001138	1.263928×10^{-5}	0.002846273	7.964667×10^{-7}	9.583692×10^{-7}
	Different TE environment	rho	**0.55***	**0.26***	**0.30***	**0.56***	**0.39***	**0.34***	**0.56***	**0.28***	**0.28***	**0.55***	**0.25***	**0.23***
		q value	2.732265×10^{-5}	0.005236055	1.602788×10^{-16}	1.95594E-05	1.952677×10^{-5}	6.052478×10^{-21}	1.557684×10^{-5}	0.002929326	2.840376×10^{-15}	2.582694×10^{-5}	0.007130352	1.66756×10^{-10}
		rho	**0.55***	**0.38***	**0.29***	**0.57***	**0.46***	**0.35***	**0.55***	**0.41***	**0.28***	**0.51***	**0.38***	**0.25***

	All TE environment	*q* value	2.134603×10^{-8}	2.363427×10^{-7}	2.387634×10^{-22}	4.248289×10^{-9}	1.096272×10^{-10}	1.991005×10^{-32}	1.41108×10^{-8}	2.692403×10^{-8}	4.567414×10^{-20}	2.965424×10^{-7}	2.541055×10^{-7}	1.897055×10^{-16}
H3K4me1	Same TE environment	rho	**0.57***	**0.53***	0.06	**0.55***	**0.46***	**0.14***	**0.38***	**0.43***	0.10	**0.39***	**0.51***	**0.13***
		q value	*0.0001144311*	8.85315×10^{-6}	*0.2620901*	*0.00028141*	*0.0001915401*	*0.01536586*	*0.01604542*	*0.0005727606*	*0.07696728*	*0.01165214*	1.957557×10^{-5}	*0.02038169*
	Different TE environment	rho	**0.37***	**0.20***	**0.15***	**0.43***	**0.43***	**0.21***	**0.36***	**0.25***	**0.14***	**0.33***	**0.39***	**0.17***
		q value	*0.007661365*	*0.03356379*	3.530144×10^{-5}	*0.001442349*	*0.000002554*	1.383879×10^{-8}	*0.008983329*	*0.008549336*	9.534421×10^{-5}	*0.01676356*	1.957557×10^{-5}	6.944531×10^{-6}
	All TE environment	rho	**0.45***	**0.35***	**0.13***	**0.50***	**0.47***	**0.19***	**0.39***	**0.33***	**0.13***	**0.35***	**0.44***	**0.16***
		q value	8.85315×10^{-6}	3.056777×10^{-6}	3.074388×10^{-5}	4.367357×10^{-7}	5.160549×10^{-11}	6.837878×10^{-10}	*0.0001145735*	7.732592×10^{-6}	1.950978×10^{-5}	*0.0006335817*	1.145595×10^{-9}	4.417853×10^{-7}

*****statistically significant correlations (*q* values < 0.05). In green are represented activating histone modifications and in red the repressing histone modifications.

Supplementary table S5: Correlations of the methylation level between duplicated genes from a same family, according to the age of the family and the TE neighborhood

		All TE environment		Same TE environment		Different TE environment	
		Rho	*q* value	Rho	*q* value	Rho	*q* value
cd14+cd16-	Young	**0.41***	0.0006829208	**0.50***	0.0045651136	**0.31***	0.0354232052
	Middle age	**0.29***	0.0007511711	**0.36***	0.0078292314	**0.23***	0.0222654948
	Old	**0.067***	0.0357849120	0.06	0.2771158371	0.07	0.0742117801
erythroblast	Young	**0.45***	0.0002131884	**0.40***	0.0275116412	**0.49***	0.0007533721
	Middle age	**0.31***	0.0002131884	**0.40***	0.0029190721	**0.26***	0.0078292314
	Old	**0.09***	0.0033609788	**0.13***	0.0222654948	**0.076***	0.0357849120
macrophage	Young	**0.43***	0.0003063971	**0.53***	0.0033376305	**0.35***	0.0144929323
	Middle age	**0.26***	0.0007533721	**0.38***	0.0033609788	**0.19***	0.0370875568
	Old	**0.12***	0.0004379365	**0.20***	0.0007533721	**0.08***	0.0308155107
cd8T	Young	**0.42***	0.0006829208	**0.51***	0.0078292314	**0.36***	0.0144929323
	Middle age	**0.33***	0.0001427133	**0.40***	0.0024162129	**0.28***	0.0036864784
	Old	0.08	0.0123890581	**0.12***	0.0327223886	0.06	0.1073429742

*****statistically significant correlations (*q* values < 0.05)

Supplementary table S6: Correlations of methylation level or histone enrichment of the duplicated genes according to the level of expression divergence between the two genes across all tissues

	**Same TE environment**				**Different TE environment**			
	**Level of expression divergence**							
	Very low	**Low**	**Medium**	**High**	**Very low**	**Low**	**Medium**	**High**
H3K4me1	**0.42* (4.457600 × 10^{-4})**	0.17 (1.736431 × 10^{-1})	0.18 (2.595250 × 10^{-1})	-0.06 (7.716800e × 10^{-1})	0.15 (9.156000 × 10^{-2})	**0.14*** (4.032000 × 10^{-2})	0.11 (2.620121 × 10^{-1})	**0.26* (3.397333× 10^{-2})**
H3K4me3	**0.57* (1.772960 × 10^{-7})**	**0.29* (8.163077 × 10^{-3})**	0.20 (2.174000 × 10^{-1})	-0.32 (1.057600 × 10^{-1})	**0.33* (5.226667 × 10^{-5})**	**0.20* (3.164622e-03)**	0.07 (5.029405 × 10^{-1})	0.02 (8.888151 × 10^{-1})
H3K27ac	**0.50* (1.766240 × 10^{-5})**	0.10 (4.673600 × 10^{-1})	0.01 (9.393481 × 10^{-1})	0.13 (5.594400 × 10^{-1})	**0.29* (4.650800 × 10^{-4})**	0.11 (1.382500 × 10^{-1})	-0.04 (7.252000 × 10^{-1})	0.05 (7.531404× 10^{-1})
H3K36me3	**0.39* (1.034400 × 10^{-3})**	**0.29* (8.163077 × 10^{-3})**	0.03 (8.379538 × 10^{-1})	**0.27* (3.552080 × 10^{-3})**	**0.25* (3.164622 × 10^{-3})**	0.05 (5.457846 × 10^{-1})	**-0.20* (2.361450 × 10^{-2})**	-0.18 (1.736431 × 10^{-1})
H3K27me3	**0.41* (4.650800 × 10^{-4})**	0.12 (3.460471 × 10^{-1})	-0.08 (6.674667 × 10^{-1})	-0.01 (9.708000× 10^{-1})	**0.19* (3.103388 × 10^{-2})**	0.09 (2.482667 × 10^{-1})	-0.05 (6.752558 × 10^{-1})	0.05 (7.485739 × 10^{-1})
H3K9me3	**0.31* (1.276400 × 10^{-2})**	**0.26* (2.088800 × 10^{-2})**	0.06 (7.485739 × 10^{-1})	-0.11 (6.476878 × 10^{-1})	0.03 (7.563500 × 10^{-1})	0.10 (1.794489 × 10^{-1})	-0.03 (7.877333 × 10^{-1})	0.01 (9.708000 × 10^{-1})
Methylation level	**0.33* (6.877818 × 10^{-3})**	0.15 (2.276690 × 10^{-1})	-0.05 (7.602286 × 10^{-1})	-0.16 (4.910889 × 10^{-1})	0.14 (1.325009 × 10^{-1})	0.08 (2.595250 × 10^{-1})	0.07 (5.247789 × 10^{-1})	0.20 (1.325009 × 10^{-1})

*****statistically significant correlations (*q* values < 0.05); *q* values are indicated in parenthesis. In red are indicated repressive epigenetic modifications and in green activating epigenetic modifications

Supplementary Table S7: Correlation of the histone enrichment between genes from a same young family, according to the TE neighborhood and the position on chromosome

			**Macrophage**		**Erythroblast**		**cd14+cd16-**		**cd8T**	
			Same chromosome	Different chromosome	Same chromosome	Different chromosome	Same chromosome	Different chromosome	Same chromosome	Different chromosome
H3K36me3	Same TE environment	rho	**0.70***	0.61	**0.75***	0.54	**0.78***	0.02	**0.67***	-0.23
		q value	9.896000×10^{-5}	7.937561×10^{-2}	1.339733×10^{-5}	1.491927×10^{-1}	5.292000×10^{-6}	9.898435×10^{-1}	2.364988×10^{-4}	7.024762×10^{-1}
	Different TE environment	rho	0.03	0.16	**0.45***	0.06	**0.47***	0.05	**0.49***	-0.04
		q value	6.415200×10^{-2}	7.024762×10^{-1}	2.874667×10^{-2}	9.081831×10^{-1}	1.878034×10^{-2}	9.205714×10^{-1}	1.444800×10^{-2}	9.423273×10^{-1}
H3K9me3	Same TE environment	rho	0.12	0.30	**0.53***	**0.74***	-0.08	0.10	-0.08	**0.74***
		q value	7.639059×10^{-1}	6.332377×10^{-1}	7.241143×10^{-3}	1.716988×10^{-2}	8.385730×10^{-1}	8.964293×10^{-1}	8.385730×10^{-1}	1.716988×10^{-2}
	Different TE environment	rho	0.41	-0.11	-0.01	0.006	0.12	-0.08	0.34	-0.25
		q value	5.371077×10^{-2}	8.007211×10^{-1}	9.898435×10^{-1}	9.996255×10^{-1}	7.630545×10^{-1}	8.792842×10^{-1}	1.328819×10^{-1}	4.619755×10^{-1}
H3K4me1	Same TE environment	rho	**0.69***	0.21	**0.59***	0.33	**0.61***	-0.06	**0.52***	0
		q value	1.107429×10^{-4}	7.630545×10^{-1}	2.129600×10^{-3}	5.640000×10^{-1}	1.108174×10^{-3}	9.423273×10^{-1}	8.169931×10^{-3}	*1.000000*
	Different TE environment	rho	**0.64***	-0.07	**0.69***	-0.11	**0.57***	0.04	**0.63***	-0.17
		q value	4.285714×10^{-4}	8.883600×10^{-1}	1.098092×10^{-4}	8.017333×10^{-1}	3.019008×10^{-3}	9.423273×10^{-1}	6.026182×10^{-4}	6.825931×10^{-1}
H3K4me3	Same TE environment	rho	**0.67***	0.11	**0.66***	0.19	**0.77***	-0.10	**0.66***	-0.07
		q value	2.364988×10^{-4}	8.883600×10^{-1}	3.035040×10^{-4}	7.707429×10^{-1}	6.974400×10^{-6}	8.964293×10^{-1}	3.035040×10^{-4}	9.322165×10^{-1}
	Different TE environment	rho	**0.76***	0.17	**0.77***	0.18	**0.82***	0.16	**0.87***	-0.006
		q value	1.048046×10^{-5}	6.825931×10^{-1}	6.974400×10^{-6}	6.756000×10^{-1}	6.166400×10^{-7}	7.024762×10^{-1}	1.092960×10^{-8}	9.996255×10^{-1}
H3K27me3	Same TE environment	rho	**0.42***	**1***	**0.56***	-0.13	**0.75***	0.60	**0.57***	0
		q value	4.802526×10^{-2}	2.112000×10^{-14}	3.493333×10^{-3}	8.670720×10^{-1}	1.339733×10^{-5}	8.555429×10^{-2}	3.302769×10^{-3}	*1.000000*
	Different TE environment	rho	0.14	-0.25	0.19	0.27	**0.65***	0.23	-0.07	-0.16
		q value	7.024762×10^{-1}	4.493617×10^{-1}	5.640000×10^{-1}	3.925565×10^{-1}	3.035040×10^{-4}	5.063040×10^{-1}	8.883600×10^{-1}	7.024762×10^{-1}
H3K27ac	Same TE environment	rho	0.11	0.21	0.26	-0.05	**0.50***	-0.11	**0.51***	0.03
		q value	7.639059×10^{-1}	7.534500×10^{-1}	3.281067×10^{-1}	9.455461×10^{-1}	1.301265×10^{-2}	8.883600×10^{-1}	1.068160×10^{-2}	9.808000×10^{-1}
	Different TE environment	rho	**0.71***	-0.12	**0.71***	0.18	**0.68***	0.18	**0.43***	-0.25
		q value	6.768873×10^{-5}	7.707429×10^{-1}	5.750400×10^{-5}	6.756000×10^{-1}	1.332480×10^{-4}	6.756000×10^{-1}	3.821838×10^{-2}	4.619755×10^{-1}

*****statistically significant correlations (*q* values < 0.05). In green are represented activating histone modifications and in red the repressing histone modifications. In total, 62 pairs of genes are located on the same chromosome (31 with different TE environment and 31 with the same TE environment) and 37 pairs of genes are located on different chromosomes (25 with different TE environment and 12 with the same TE environment).
